# A nonparametric framework for inferring orders of categorical data from category-real pairs

**DOI:** 10.1016/j.heliyon.2020.e05435

**Published:** 2020-11-06

**Authors:** Chainarong Amornbunchornvej, Navaporn Surasvadi, Anon Plangprasopchok, Suttipong Thajchayapong

**Affiliations:** National Electronics and Computer Technology Center (NECTEC), Pathum Thani, 12120, Thailand

**Keywords:** Computer Science, Ordering inference, Estimation statistics, Bootstrapping, Nonparametric method, Data Science, Income inequality

## Abstract

Given a dataset of careers and incomes, how large a difference of incomes between any pair of careers would be? Given a dataset of travel time records, how long do we need to spend more when choosing a public transportation mode *A* instead of *B* to travel? In this paper, we propose a framework that is able to infer orders of categories as well as magnitudes of difference of real numbers between each pair of categories using an estimation statistics framework. Our framework not only reports whether an order of categories exists, but it also reports magnitudes of difference of each consecutive pair of categories in the order. In a large dataset, our framework is scalable well compared with existing frameworks. The proposed framework has been applied to two real-world case studies: 1) ordering careers by incomes from 350,000 households living in Khon Kaen province, Thailand, and 2) ordering sectors by closing prices from 1,060 companies in NASDAQ stock market between years 2000 and 2016. The results of careers ordering demonstrate income inequality among different careers. The stock market results illustrate dynamics of sector domination that can change over time. Our approach is able to be applied in any research area that has category-real pairs. Our proposed *Dominant-Distribution Network* provides a novel approach to gain new insight of analyzing category orders. A software of this framework is available for researchers or practitioners in an R CRAN package: EDOIF.

## 1 Introduction

We use an order of items with respect to their specific properties all the time to make our decision. For instance, when we plan to buy a new house, we might use an ordered list of houses based on their prices or distances from a downtown. We might use travel times to order a list of transportation modes to decide which option is the best to travel from A to B, etc.

Ordering is related to a concept of *partial order* or poset in order theory [1]. A well-known form of poset is a directed acyclic graph (DAG) that is widely used in studying of causality [2], [3], animal behavior [4], social networks [5], [6], etc. Additionally, in social science, ordering of careers based on incomes can be applied to a study of inequality in society (see Section 7.2).

Hence, ordering is an important concept that is used daily and can impact society decision and scientific research. However, in the era of big data, inferring orders of categorical items based on their real-valued properties from large datasets are far from trivial.

In this paper, we investigate a problem of inferring an order of categories based on their real-valued properties, Dominant-distribution ordering inference problem, using the poset concept [1] as well as estimating a magnitude of difference between any pair of categories. We also propose a *Dominant-Distribution Network* as a representation of dominant category orders. We develop our framework based on a new concept of statistics named *Estimation Statistics* principle. The aim of estimation statistics is to resolve issues of the traditional methodology, null hypothesis significance testing (NHST), that focuses on using p-value to make a dichotomous yes-no question (see Section 2).

In an aspect of scalability, our framework can finish analyzing a dataset of 10,000 data points in 11 seconds while a candidate approach needs 300 seconds for the same dataset. The software of our proposed framework is available for researchers and practitioners with a user-friendly R CRAN package: EDOIF at [7].

This paper is organized as follows. Section 2 reviews related works, analyzing existing gaps and how our contributions address them. Then, Section 5 describes our proposed framework. Experimental setup is shown in Section 6 where corresponding results are discussed in Section 7. Finally, Section 8 concludes this paper.

**Figure:**
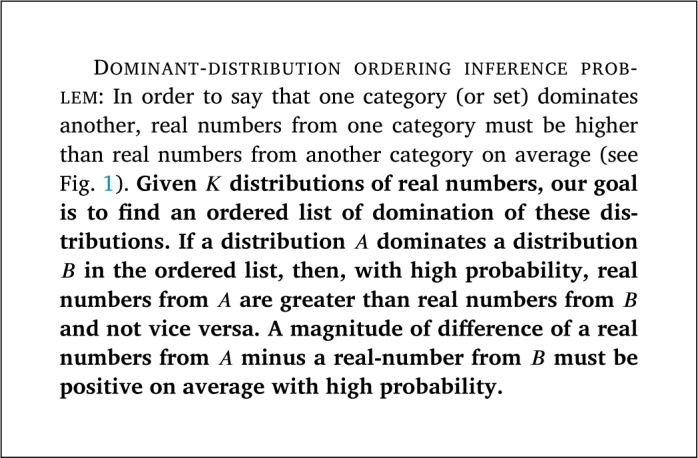

## 2 Related works

There are several NHST frameworks in both parametric (e.g. Student's t-test [8]) and nonparametric (Mann-Whitney test [9]) types that are able to compare two distributions and report whether one has a greater sample mean or median than another using a p-value. Nevertheless, these approaches are not capable of providing a magnitude of mean difference between two distributions. Moreover, there are several issues of using only p-values to compare distributions. For instance, a null hypothesis might always get rejection since, in some system, there is always some effect but an effect might be too small [10]. The NHST also treats distribution comparison as a dichotomous yes-no question and ignores a magnitude of difference, which might be an important information for a research question [11]. Besides, using only a p-value information is a major issue on repeatability in many research publications [12].

Hence, *Estimation Statistics* has been developed as an alternative methodology to NHST. The estimation statistics is considered to be more informative than NHST [13], [14], [15]. A primary purpose of estimation-statistic methods is to determine magnitudes of difference among distributions in terms of point estimates and confidence intervals rather than reporting only a p-value in NHST.

**Figure 1:**
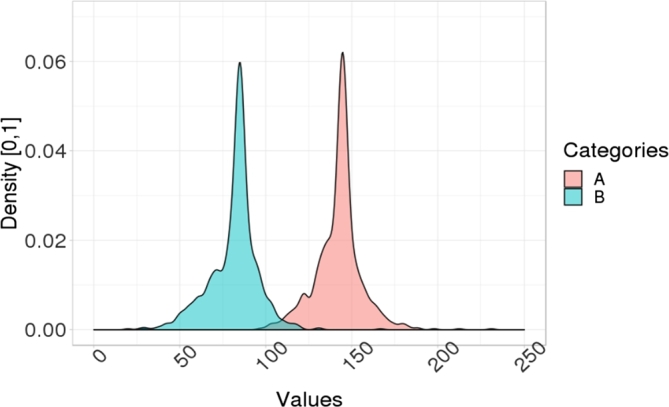
An example of distribution of category *A* dominates a distribution of category *B*. A probability of a data point *a* in *A* s.t. *a* ≥ *E*[*B*] is greater than a probability of a data point *b* in *B* s.t. *b* ≥ *E*[*A*].

**Figure 2:**
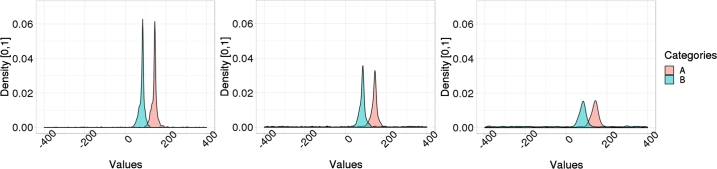
An example of distribution of category *A* dominates distribution of category *B* with different degrees of uniform noise w.r.t. total data density: (left) 1%, (middle) 20%, and (right) 40% of noise. A higher degree of uniform noise implies that it is harder to distinguish whether *A* dominates *B*.

Recently, the Data Analysis using Bootstrap-Coupled ESTimation in R (DABESTR) framework [15], which is an estimation-statistics approach, has been developed. It mainly uses Bias-corrected and accelerated (BCa) bootstrap [16] as a main approach to estimate a confidence interval of mean difference between distributions. BCa bootstrap is robust against a skew issue in a distribution [16] than a percentile confidence interval and other approaches. However, it is not obvious whether BCa bootstrap is better than other approaches in the task of inferring a confidence interval of mean difference when two distributions have a high level of uniform noise (see Fig. 2). Moreover, DABESTR is not scalable well when there are many pairs of distributions to compare; it cannot display all confidence intervals of mean difference of all pairs in a single plot. Another issue of using BCa bootstrap is that it is too slow (see Section 6.5) in practice compared to other approaches. There is also no problem formalization of Dominant-distribution ordering inference problem, which should be considered as a problem that can be formalized by the *Order Theory*, using a partial order concept [1].

### 2.1 Our contributions

To fill these gaps in the field, we formalize Dominant-distribution ordering inference problem using a partial order concept [1] in the order theory (see Section 3). We provide a framework as a solution of Dominant-distribution ordering inference problem. Our framework is a non-parametric framework based on a bootstrap principle that has no assumption regarding models of data (see Section 4). We also propose a representation for a dominant order namely *Dominant-Distribution Network* (Definition 4). Our proposed framework is capable of:

• **Inferring an order of multiple categories:** inferring orders of domination of categories and representing orders in a graph form;
• **Estimating a magnitude of difference between a pair of categories:** estimating confidence intervals of mean difference for all pairs of categories; and
• **Visualizing a network of dominant orders and magnitudes of difference among categories:** visualizing dominant orders in one graph entitled, *Dominant-Distribution Network*, as well as illustrating all magnitudes of difference of all category pairs within a single plot.

We evaluate our framework in an aspect of sensitivity analysis of uniform noise using simulation datasets that we posses a ground truth to compare our framework against several methods. To demonstrate real-world applications of our framework, we also provide two case studies. The first is a case of inferring income orders of careers in order to measure income inequality in Khon Kaen province, Thailand based on surveys of 350,000 households. Another case study is to use our framework to study dynamics of sector domination in NASDAQ stock market using 1,060 companies stock-closing prices between 2000 and 2016. The assessment on these two independent/irrelevant domains indicates the potential that our framework is applicable to any field of study that requires ordering of categories based on real-valued data. Our *Dominant-Distribution Network* (Definition 4) provides a novel approach to gain insight of analyzing category orders.

### 2.2 Why confidence intervals?

We can simply order categories by their means or medians. However, comparing only means cannot tell us how much overlapping areas of values from two categories are. Hence, we need mean confidence intervals to approximate overlapping areas as well as using mean-difference confidence intervals to tell a magnitude of difference between two categories. Additionally, if there are many categories and we want to infer how many pairs of categories dominate others, then we can use a network to represent these dominant relationships. In this paper, we propose a network called a *Dominant-distribution network* to represent dominant relationships among categories.

## 3 Problem formalization

In this section, we provide details regarding that a dominant-distribution relation is a partial order as well as providing the problem formalization of Dominant-distribution ordering inference problem.

For any given pair of categories A,B, we define an order that category *A* dominates category *B* using their real random variables as follows.

Definition 1 **Dominant-distribution relation**

Given two continuous random variables $X_{1}∼D_{1}$ and $X_{2}∼D_{2}$ where $D_{1},D_{2}$ are distributions. Assuming that $D_{1}$ and $D_{2}$ have the following property: $P(X_{1}\geq E[X_{1}])=P(X_{2}\geq E[X_{2}])$. We say that $D_{2}$ dominates $D_{1}$ if $P(X_{1}\geq E[X_{2}])\leq P(X_{2}\geq E[X_{1}])$; denoting $D_{1}⪯D_{2}$. We denote $D_{1}≺D_{2}$ if $P(X_{1}\geq E[X_{2}])<P(X_{2}\geq E[X_{1}])$.

We provide a concept of equivalent distributions as follows.

Proposition 3.1 *Let* $D_{1},D_{2}$ *be distributions such that* $D_{1}⪯D_{2}$ *and* $D_{2}⪯D_{1}$*, then* $D_{1},D_{2}$ *are equivalent distributions denoted* $D_{1}\equiv D_{2}$*.*

Proof When $D_{1}⪯D_{2}$ and $D_{2}⪯D_{1}$, the first obvious case is $P(X_{1}\geq E[X_{2}])=P(X_{2}\geq E[X_{1}])$. For the case that $D_{1}≺D_{2}$ and $D_{2}≺D_{1}$, this cannot happen because of contradiction. Hence, $D_{1}⪯D_{2}$ and $D_{2}⪯D_{1}$ implies only $P(X_{1}\geq E[X_{2}])=P(X_{2}\geq E[X_{1}])$. □

We provide a relationship between expectations of distribution and a dominant-distribution relation below.

Proposition 3.2 *Let* $D_{1},D_{2}$ *be distributions, and* $X_{1}∼D_{1},X_{2}∼D_{2}$ *s.t.* $P(X_{1}\geq E[X_{1}])=P(X_{2}\geq E[X_{2}])$*.* $E[X_{1}]\leq E[X_{2}]$ *if and only if* $D_{1}⪯D_{2}$*.*

Proof In the forward direction, suppose $E[X_{1}]\leq E[X_{2}]$. Because the center of $D_{2}$ is on the right of $D_{1}$ in the real-number axis, hence, $P(X_{2}\geq E[X_{1}])$ covers most areas of $D_{2}$ distribution except the area of $P(X_{2}<E[X_{1}])$. In contrast, $P(X_{1}\geq E[X_{2}])$ covers only a tiny area in the far right of $D_{1}$. This implies that $P(X_{1}\geq E[X_{2}])\leq P(X_{2}\geq E[X_{1}])$ or $D_{1}⪯D_{2}$.

In the backward direction, we use the proof by contradiction. Suppose $D_{1}⪯D_{2}$. Because $D_{1}⪯D_{2}$ implies $P(X_{1}\geq E[X_{2}])\leq P(X_{2}\geq E[X_{1}])$ and $P(X_{1}\geq E[X_{1}])=P(X_{2}\geq E[X_{2}])$, then we have the following implications.

Let us assume that $E[X_{2}]<E[X_{1}]$. This implies that $P(X_{1}\geq E[X_{1}])<P(X_{1}\geq E[X_{2}])$. Since $P(X_{1}\geq E[X_{1}])=P(X_{2}\geq E[X_{2}])$, we have

(1) $$P(X_{2}\geq E[X_{2}])<P(X_{1}\geq E[X_{2}]).$$

Assuming $E[X_{2}]<E[X_{1}]$, we also have

(2) $$P(X_{2}\geq E[X_{1}])<P(X_{2}\geq E[X_{2}]).$$

By combining inequation (1) and inequation (2), we have

(3) $$P(X_{2}\geq E[X_{1}])<P(X_{1}\geq E[X_{2}]).$$

The inequation (3) contradicts with the requirement of $D_{1}⪯D_{2}$, which is $P(X_{1}\geq E[X_{2}])\leq P(X_{2}\geq E[X_{1}])$! Therefore, $E[X_{1}]\leq E[X_{2}]$. □

In the next step, we show that a dominant-distribution relation has a transitivity property.

Proposition 3.3 *Let* $D_{1},D_{2},D_{3}$ *be distributions such that* $D_{1}⪯D_{2}$*,* $D_{2}⪯D_{3}$*, then* $D_{1}⪯D_{3}$*.*

Proof According to Proposition 3.2, $D_{1}⪯D_{2}$ implies $E[X_{1}]\leq E[X_{2}]$.

Now, we have $E[X_{1}]\leq E[X_{2}]\leq E[X_{3}]$. The $D_{3}$ distribution must be on the right hand side of $D_{1}$. Hence, $P(X_{1}\geq E[X_{3}])\leq P(X_{3}\geq E[X_{1}])$, which implies $D_{1}⪯D_{3}$. □

Now, we are ready to conclude that a dominant-distribution relation is a partial order on a set of continuous distributions.

Theorem 3.4 *Given a set S of continuous distributions s.t. for any pair* $D_{1},D_{2}\in S$*. Assuming that for any* $X_{1}∼D_{1},X_{2}∼D_{2}$*,* $P(X_{1}\geq E[X_{1}])=P(X_{2}\geq E[X_{2}])$*. The* dominant-distribution relation *is a partial order on a set S* *[1]**.*

Proof A relation is a partial order on a set *S* if it has the following properties: antisymmetry, transitivity, and reflexivity.

• **Antisymmetry:** if $D_{1}⪯D_{2}$ and $D_{2}⪯D_{1}$, then $D_{1}\equiv D_{2}$ by Proposition 3.1.
• **Transitivity:** if $D_{1}⪯D_{2}$, $D_{2}⪯D_{3}$, then $D_{1}⪯D_{3}$ by Proposition 3.3.
• **Reflexivity:** ∀*D*, D⪯D.

Therefore, by definition, the dominant-distribution relation is a partial order on a set of continuous distributions. □

Suppose we have $D_{1}⪯D_{2}$ and $X_{1}∼D_{1},X_{2}∼D_{2}$. We can have $Y=X_{2}-X_{1}$ as a random variable that represents a magnitude of difference between two distributions. Suppose $\mu_{Y}$ is the true mean of *Y*'s distribution, our next goal is to find the confidence interval of $\mu_{Y}$.

Definition 2 ***α*-Mean-difference confidence interval**

Given two continuous random variables $X_{1}∼D_{1}$ and $X_{2}∼D_{2}$ where $D_{1},D_{2}$ are distributions, $Y=X_{2}-X_{1}$, and α∈[0,1]. An interval [l,u] is *α*-mean-difference confidence interval if $P(l\leq \mu_{Y}\leq u)\geq 1-\alpha$.

Now, we are ready to formalize Dominant-distribution ordering inference problem.

**Problem 1:**
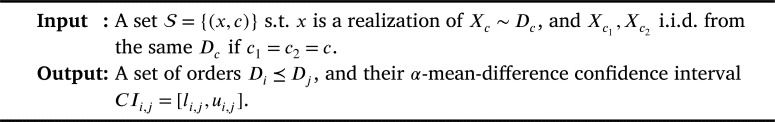
Dominant-distribution ordering inference problem.

## 4 Statistical inference

### 4.1 Bootstrap approach

Suppose we have $Y=X_{2}-X_{1}$ and $Y∼D_{Y}$ with the unknown $\mu_{Y}$, we can use the mean $\overset{¯}{Y}=E[Y]$ as the point estimate of $\mu_{Y}$ since it is an unbiased estimator. We deploy the estimation statistics [13], [14], [15], which is a framework that focuses on estimating an effect sizes, *Y*, of two distributions. Compared to null hypothesis significance testing approach (NHST), estimation statistics framework reports not only whether two distributions are significantly different, but it also reports magnitudes of difference in the form of confidence interval.

The estimation statistics framework uses bootstrap technique [17] to approximately infer a bootstrap confidence interval of $\mu_{Y}$. Assuming that the number of times of bootstrapping is large, according to the Central Limit Theorem (CLT), even though an underlying distribution is not normal distributed, summary statistics (e.g. means) of random sampling approaches a normal distribution. Hence, we can use a normal confidence interval to approximate the confidence interval of $\mu_{Y}$.

Theorem 4.1 **Central Limit Theorem (CLT) [18]**

*Given* $X_{1},\ldots ,X_{n}$ *be i.i.d. random variables with* $E[X_{i}]=\mu <\infty$ *and* $0<\text{VAR}(X_{i})=\sigma^{2}<\infty$*, and* $\overset{¯}{X}=\frac{\sum_{i=1}^{n}X_{i}}{n}$*. Then, the random variable*

$$Z_{n}=\frac{\overset{¯}{X}-\mu }{\sigma /\sqrt{n}}$$

*converges in distribution to a standard normal random variable as n goes to infinity, that is*

$$\lim_{n\to \infty }P(Z_{n}\leq x)=\Phi (x),\forall x\in R,$$

*where* Φ(x) *is the standard normal CDF.*

Lemma 4.2 *Given* $X_{1,1},\ldots ,X_{1,k}$ *are random variables i.i.d. from* $D_{1}$*,* $X_{2,1},\ldots ,X_{2,k}$ *are random variables i.i.d. from* $D_{2}$*, and* $Y_{1},\ldots ,Y_{k}$ *are random variables where* $Y_{i}=X_{2,i}-X_{1,i}$*.*

*Assuming that the number k is large, the distribution of* $Y_{i}$ *is unknown with an unknown variance* $\text{VAR}(Y_{i})=\sigma_{Y}^{2}<\infty$*. Suppose* $\overset{¯}{Y}$ *is the sample mean of* $Y_{1},\ldots ,Y_{k}$*,* $\mu_{Y}=E[Y_{i}]$*, and* $s_{Y}$ *is their standard deviation. Given that* Φ(⋅) *is the standard normal CDF and* $z_{\frac{\alpha }{2}}=\Phi^{-1}(1-\frac{\alpha }{2})$*, then the interval*

(4) $$CI_{\overset{¯}{Y}}=[\overset{¯}{Y}-z_{\frac{\alpha }{2}}\frac{s_{Y}}{\sqrt{k}},\overset{¯}{Y}+z_{\frac{\alpha }{2}}\frac{s_{Y}}{\sqrt{k}}]$$

*is approximately* (1−α)100% *confidence interval for* $\mu_{Y}$*.*

Proof Since *k* is large, the distribution of sample mean of $Y_{1},\ldots ,Y_{k}$ follows the Central Limit Theorem. This implies that the random variable

$$Z_{k}=\frac{\overset{¯}{Y}-\mu_{Y}}{\sigma_{Y}/\sqrt{k}}$$

has approximately N(0,1) distribution. Hence, $\overset{¯}{Y}$ is approximately normal distributed from $N(\mu_{Y},\sigma_{Y}/\sqrt{k})$. The (1−α)100% confidence interval for $\overset{¯}{Y}$ is $\left[\mu_{Y}-z_{\frac{\alpha }{2}}\frac{\sigma_{Y}}{\sqrt{k}},\mu_{Y}+z_{\frac{\alpha }{2}}\frac{\sigma_{Y}}{\sqrt{k}}\right]$.

Since $\overset{¯}{Y}$ is the unbiased estimator of $\mu_{Y}$ and $s_{Y}$ is the unbiased estimator of $\sigma_{Y}$, we can have the approximation of (1−α)100% confidence interval of $\mu_{Y}$ as follows.

$$\left[\overset{¯}{Y}-z_{\frac{\alpha }{2}}\frac{s_{Y}}{\sqrt{k}},\overset{¯}{Y}+z_{\frac{\alpha }{2}}\frac{s_{Y}}{\sqrt{k}}\right]$$

 □

According to Lemma 4.2, we need to access to a large number of $Y_{1},\ldots ,Y_{k}$ to infer the confidence interval. We can generate $Y_{1},\ldots ,Y_{k}$ s.t. *k* is large using a bootstrap technique. The following theorem allows us to approximate the mean of $Y_{i}$ in a bootstrap approach.

Theorem 4.3 **Bootstrap convergence [19], [20]**

*Given* $X_{1},\ldots ,X_{n}$ *are random variables i.i.d. from an unknown distribution D with* $\text{VAR}(X_{i})=\sigma^{2}<\infty$*. We choose* $X_{1}^{\prime },\ldots ,X_{m}^{\prime }$ *from the set* $\left\{X_{1},\ldots ,X_{n}\right\}$ *by resampling with replacement. As* n,m *approach* ∞*:*

• **Asymptotic mean:** *a conditional distribution of* $\sqrt{m}({\overset{¯}{X}}^{\prime }-\overset{¯}{X})$ *given* $X_{1},\ldots ,X_{n}$ *converges weakly to* $N(0,\sigma^{2})$*.*
• **Asymptotic standard deviation:** $s_{m}\overset{}{\to }\sigma$ *in a conditional probability: that is for any positive ϵ,*
  $$P(|s_{m}-\sigma |>\epsilon |X_{1},\ldots ,X_{n})\overset{}{\to }0,$$
  *where* ${\overset{¯}{X}}^{\prime }=m^{-1}\sum_{1}^{m}X_{i}^{\prime }$*,* $\overset{¯}{X}=n^{-1}\sum_{1}^{n}X_{i}$*, and* $s_{m}^{2}=m^{-1}\sum_{1}^{m}{\left(X_{i}^{\prime }-{\overset{¯}{X}}^{\prime }\right)}^{2}$*.*

From Theorem 4.3, when we increase a number of times we perform the resampling with replacement on $D_{1},D_{2}$ to be large, we can approximate the $\overset{¯}{Y}$ using the bootstrap sample mean ${\overset{¯}{Y}}^{\prime }$. The same applies for the standard deviation $s_{Y}$ that we can use its bootstrap version $s_{Y}^{\prime }$ to approximate it. By using ${\overset{¯}{Y}}^{\prime },s_{Y}^{\prime }$, we can approximate the confidence interval in Lemma 4.2.

### 4.2 Dominant-distribution relation inference

According to Proposition 3.2, $E[X_{1}]\leq E[X_{2}]$ implies $D_{1}⪯D_{2}$. Suppose that $\mu_{1}=E[X_{1}]$ and $\mu_{2}=E[X_{2}]$ are also random variables. If $P(\mu_{1}\leq \mu_{2})$ or $P(\mu_{2}-\mu_{1}\geq 0)=1$, then $P(D_{1}⪯D_{2})=1$. However, in reality, $P(\mu_{2}-\mu_{1}\geq 0)$ might not equal to one due to noise. Hence, we define the following notion of a relaxing dominant-distribution relation.

Definition 3 ***α*-Dominant-distribution relation**

Given two continuous random variables $X_{1}∼D_{1}$ and $X_{2}∼D_{2}$ where $D_{1},D_{2}$ are distributions, and α∈[0,1]. Suppose $\mu_{1}=E[X_{1}],\mu_{2}=E[X_{2}]$, we say that $D_{2}$ dominates $D_{1}$ if $P(E[\mu_{2}-\mu_{1}]\geq 0)\geq 1-\alpha$; denoting $D_{1}{⪯}_{\alpha }D_{2}$.

Suppose we have two empirical distributions $D_{1}^{\prime }$ and $D_{2}^{\prime }$. From Theorem 4.3 and Lemma 4.2, we can define $X_{1}$ and $X_{2}$ as random variables from sample-mean distributions $D_{1},D_{2}$ of empirical distributions $D_{1}^{\prime }$ and $D_{2}^{\prime }$. We can get $D_{1}$ and $D_{2}$ by bootstrapping data points from $D_{1}^{\prime }$ and $D_{2}^{\prime }$. Suppose $Y=X_{2}-X_{1}$, then, we can approximate the confidence interval of $\mu_{Y}=E[Y]$ with *α* using the interval $CI_{\overset{¯}{Y}}$ in Lemma 4.2.

Next, we use (1−α)100% confidence interval of $\mu_{Y}$ to infer whether $D_{1}{⪯}_{\alpha }D_{2}$. Given $\mu_{y}=\mu_{2}-\mu_{1}$, according to the Definition 3, if $P(E[\mu_{Y}]\geq 0)\geq 1-\alpha$, then $D_{1}{⪯}_{\alpha }D_{2}$. We can approximate whether $E[\mu_{Y}]\geq 0$ with the probability 1−α by the approximate (1−α)100% confidence interval of $\mu_{Y}$: $CI_{\overset{¯}{Y}}=[\overset{¯}{Y}-z_{\frac{\alpha }{2}}\frac{s_{Y}}{\sqrt{k}},\overset{¯}{Y}+z_{\frac{\alpha }{2}}\frac{s_{Y}}{\sqrt{k}}]$. If the lower bound $\overset{¯}{Y}-z_{\frac{\alpha }{2}}\frac{s_{Y}}{\sqrt{k}}$ is greater than zero, then $P(E[\mu_{Y}]\geq 0)$ is approximately 1−α.

In the aspect of hypothesis test, determining whether $D_{1}{⪯}_{\alpha }D_{2}$ is the same as testing whether the expectation of $X_{1}∼D_{1}$ is less than the expectation of $X_{2}∼D_{2}$ where a null hypothesis is $E[X_{2}]-E[X_{1}]<0$ and an alternative hypothesis is $E[X_{2}]-E[X_{1}]\geq 0$. We can verify these two hypothesis by inferring the confidence interval of $\mu_{Y}=E[X_{2}]-E[X_{1}]$. If the lower bound of $\mu_{Y}$ is greater than zero with the probability 1−α, then we can reject the null hypothesis. Moreover, not only the confidence interval can test the null hypothesis, but it is also be able to tell us a magnitude of mean difference between $D_{1}$ and $D_{2}$. Hence, a confidence interval is more informative than the NHST approach.

Given a set of distributions $\left\{D_{1},\ldots ,D_{c}\right\}$, in this paper, we choose to represent *α*-Dominant-distribution relations using a network as follows.

Definition 4 **Dominant-distribution network**

Given a set of *c* continuous distributions $S=\{D_{1},\ldots ,D_{c}\}$ and α∈[0,1]. Let G=(V,E) be a directed acyclic graph. The graph *G* is a Dominant-distribution network s.t. a node i∈V represents $D_{i}$ and (i,j)∈E if $D_{j}{⪯}_{\alpha }D_{i}$.

In the Section 5, we discuss about the proposed framework that can infer a dominant-distribution network *G* from a set of category-real values.

## 5 Methods

For any given pair of categories A,B, based on Definition 1, we defined that a dominant-distribution relation of category *A* dominates category *B* exists if a value of *A* is higher than a value of *B* with high probability.

Since a dominant-distribution relation is a partial order relation (Theorem 3.4 in Section 3), an order always exists in any given set of category-real pairs. For each pair of categories *A* and *B*, we can use a bootstrap approach to infer whether A⪯B as well as using an inferred mean-difference confidence interval from bootstrapping to represent a magnitude of difference between *A* and *B* (see Section 4).

**Figure 3:**
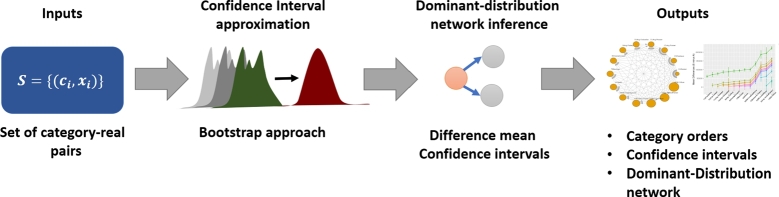
A high-level overview of the proposed framework.

We propose the Empirical Distribution Ordering Inference Framework (EDOIF) as a solution of Dominant-distribution ordering inference problem using bootstrap and additional non-parametric method. Fig. 3 illustrates an overview of our framework. Given a set of order pairs of category-real values $S=\{(c_{i},x_{i})\}$ as an input of our framework where $c_{i}\in C$ s.t. C={c} is a set of category classes, and $x_{i}\in R$, in this paper, we assume that for any pair $\left(c_{i},x_{i}),(c_{j},x_{j}\right)$ if $c_{i}=c_{j}=c$, then both $x_{i}$ and $x_{j}$ are realizations of random variables from a distribution $D_{c}^{\prime }$.

In the first step, we infer a sample-mean confidence interval of each $D_{c}^{\prime }$ and a mean-difference confidence interval between each pair of $D_{a}^{\prime }$ and $D_{b}^{\prime }$ (Section 5.1). Then, in Section 5.2, we provide details regarding the way to infer the Dominant-distribution network.

### 5.1 Confidence interval inference

We separate a set $S=\{(c_{i},x_{i})\}$ into $D_{1}^{\prime },\ldots ,D_{C}^{\prime }$ where $D_{c}^{\prime }=\{x_{i}\}$ is a set of data points $x_{i},\ldots$ that belong to a category *c* in *S*. We sort $D_{1}^{\prime },\ldots ,D_{C}^{\prime }$ based on their sample means s.t. ${\overset{¯}{X}}_{p}\leq {\overset{¯}{X}}_{p+1}$ where ${\overset{¯}{X}}_{p},{\overset{¯}{X}}_{p+1}$ are sample means of $D_{p}^{\prime },D_{p+1}^{\prime }$ respectively.

For each $D_{c}^{\prime }$, we perform the bootstrap approach (Section 4.1) to infer a sample-mean distribution $D_{c}$ and its (1−α)×100-confidence interval. Given $X_{c}∼D_{c}$ and $\mu_{c}=E[X_{c}]$, the framework infers the confidence interval of $\mu_{c}$ w.r.t. $D_{c}$ denoted $CI_{\mu_{c}}$. Algorithm 2 illustrates details on how to infer $CI_{\mu_{c}}$ using the bootstrap approach.

**Algorithm 2:**
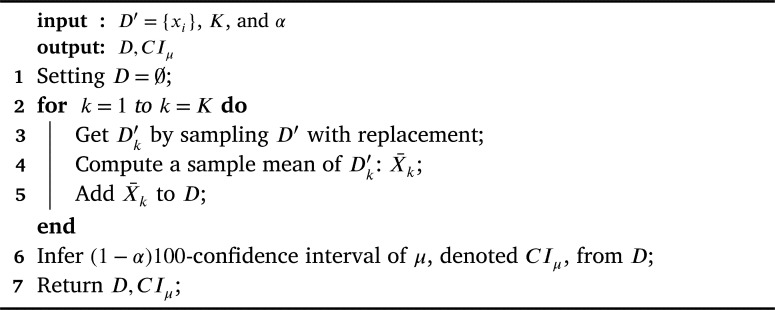
MeanBootstrapFunction.

In the next step, we infer an *α*-mean-difference confidence interval of each pair $D_{p}^{\prime },D_{q}^{\prime }$.

Given $D_{p},D_{q}$ are sample-mean distributions that are obtained by bootstrapping $D_{p}^{\prime },D_{q}^{\prime }$ respectively, $X_{p}\;∼D_{p},X_{q}∼D_{q}$, $Y=X_{q}-X_{p}$, and $\mu_{Y}=E[Y]$.

The framework uses the bootstrap approach to infer sample-mean-difference distribution of *Y* and the (1−α)100-confidence interval of $\mu_{Y}$. Algorithm 3 illustrates the details of how to infer $CI_{\overset{¯}{Y}}$ using the bootstrap approach in general.

**Algorithm 3:**
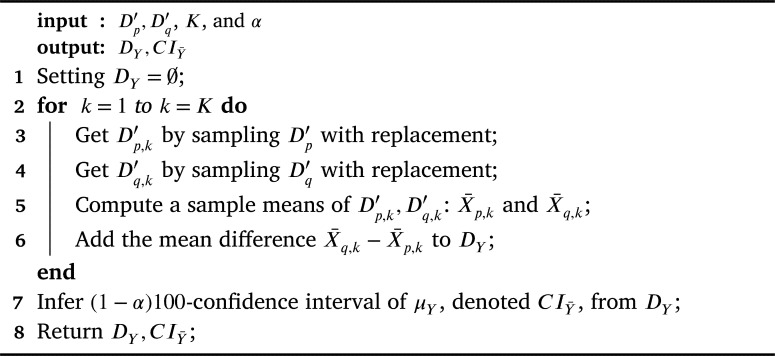
MeanDiffBootstrapFunction.

Even though we can use a normal confidence interval as a confidence interval in line 6 of Algorithm 2 and line 7 of Algorithm 3 (see Lemma 4.2), the normal bound has an issue when a distribution is skew [15], [16]. Hence, we deploy both percentile confidence intervals and Bias-corrected and accelerated (BCa) bootstrap [16] to infer both confidence intervals: $CI_{\mu_{c}}$ and $CI_{\overset{¯}{Y}}$.

For a percentile confidence interval inference (our default option) and BCa bootstrap, we deploy a standard library of bootstrap approaches in R “boot” package [21], [22], [23].

### 5.2 Dominant-distribution network inference

The first step of inferring a dominant-distribution network G=(V,E) in Definition 4 is to infer whether $D_{p}{⪯}_{\alpha }D_{q}$.

In a network G=(V,E), a node p∈V represents $D_{p}$ and (q,p)∈E if $D_{p}{⪯}_{\alpha }D_{q}$.

Given $X_{p}\;∼D_{p},X_{q}∼D_{q}$, $Y=X_{q}-X_{p}$, we can check a normal lower bound of $CI_{\overset{¯}{Y}}$ in Lemma 4.2 that we mentioned in Section 4.1. If a lower bound $\overset{¯}{Y}-z_{\frac{\alpha }{2}}\frac{s_{Y}}{\sqrt{k}}$ is greater than zero, then $D_{p}{⪯}_{\alpha }D_{q}$. However, we deploy Mann-Whitney test [9] to infer whether $D_{p}{⪯}_{\alpha }D_{q}$ due to its robustness (see Section 7). Along with Mann-Whitney test [9], we also deploy a p-value adjustment method by Benjamini and Yekutieli (2001) [24] to reduce a false positive issue.

In the next step, for each $D_{p}$, we add node *p* to *V*. For any pair $D_{p},D_{q}$, if $D_{p}{⪯}_{\alpha }D_{q}$, then (q,p)∈E. One of properties we have for *G* is that a set of nodes that are reachable by a path from *q* is a set of distributions of which $D_{q}$ dominates them.

### 5.3 Visualization

We use ggplot2 package [25] to create mean confidence intervals (e.g. Fig. 8) and mean-difference confidence intervals (e.g. Fig. 10) plots. For a dominant-distribution network, we visualize it using iGraph package [26] (e.g. Fig. 9).

## 6 Experimental setup

We use both simulation and real-world datasets to evaluate our method performance.

### 6.1 Simulation data for sensitivity analysis

We simulated datasets from a mixture distribution, which consists of normal distribution, Cauchy distribution, and uniform distribution. A random variable *X* of our mixture distribution is defined as follows.

(5) $$X∼\left\{ \begin{array}{cc} N(\mu_{0},\sigma_{0}),\; & \text{with probability }0.5\\ C(x_{0},\gamma ),\; & \text{with probability }(0.5-p_{1})\\ U(L_{1},U_{1}),\; & \text{with probability }p_{1} \end{array}\right.$$

Where $N(\mu_{0},\sigma_{0})$ is a normal distribution with mean $\mu_{0}$ and variance $\sigma_{0}^{2}$, $C(x_{0},\gamma )$ is a Cauchy distribution with location $x_{0}$ and scale *γ*, $U(L_{1},U_{1})$ is a uniform distribution with the minimum number $L_{1}$ and maximum number $U_{1}$, and $p_{1}$ is a value that represents a level of uniform noise. When the $p_{1}$ increases, the ratio of uniform distribution in the mixture distribution increases. We set $p_{1}=\{0.01,0.05,0.10,0.15,0.20,0.25,0.30,0.35,0.40\}$ to generate simulation datasets in order to perform the sensitivity analysis.

**Figure 4:**
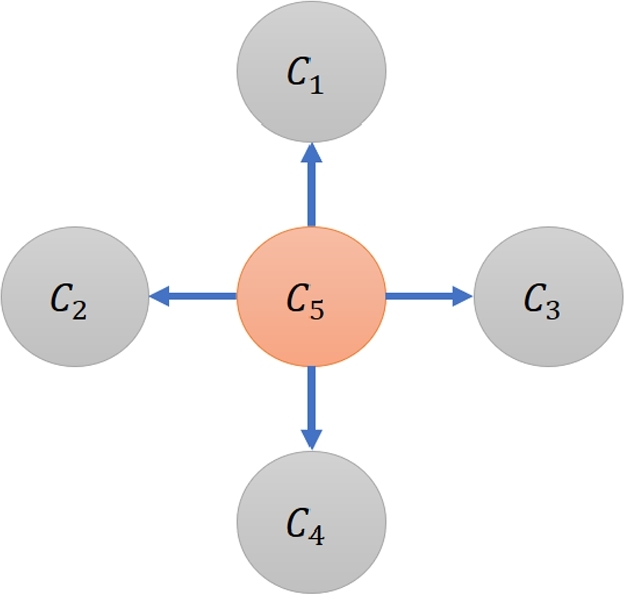
A dominant-distribution network *G* of simulation datasets.

In all simulation datasets, there are five categories: $C_{1},\ldots ,C_{5}$. The dominant-distribution relations of these categories are represented as a dominant-distribution network *G* as shown in Fig. 4 where only $C_{5}$ dominates others. For $C_{1},\ldots ,C_{4}$, we set $\mu_{0}=80,\sigma_{0}=16,x_{0}=85,\gamma =2,L_{1}=-400,U_{1}=400$ to generate realizations of *X*. For $C_{5}$, we set $\mu_{0}=140,\sigma_{0}=16,x_{0}=145,\gamma =2,L_{1}=-400,U_{1}=400$.

Because a uniform distribution in the mixture distribution has a range between -400 and 400, but all areas of distributions of $C_{1},\ldots ,C_{5}$ are within [−400,400], a method has more issue to distinguish whether $C_{i}⪯C_{j}$ for any $C_{i},C_{j}\in \{C_{1},\ldots ,C_{5}\}$ when we increase $p_{1}$ (see Fig. 2).

The main task of inference here is to measure whether a given method can infer that $C_{i}⪯C_{j}$ w.r.t. a network in Fig. 4 from these simulation datasets. We generate 100 datasets for each different value of $p_{1}$. In total, there are 900 datasets.

To measure the performance of ordering inference, we define true positive (TP), false positive (FP), and false negative (FN) in order to calculate precision, recall, and F1 score as follows. Given any pair of categories $C_{i},C_{j}$, TP is when both ground truth (Fig. 4) and inferred result agree that $C_{i}⪯C_{j}$ is true. FP is when a method infers that $C_{i}⪯C_{j}$ but the ground truth disagrees. FN is when the ground truth has $C_{i}⪯C_{j}$ but an inferred result from the method disagrees.

In the task of inferring whether $C_{i}⪯C_{j}$, we compared our approach (Mann-Whitney test [9] with p-value adjustment method [24]) against 1) t-test with Pooled Standard Deviation [27], 2) t-test with p-value adjustment [24], 3) BCa bootstrap, and 4) percentile bootstrap (Perc). For both BCa bootstrap, and percentile bootstrap, we decide whether $C_{i}⪯C_{j}$ based on the lower bound of confidence intervals of mean difference between $C_{i}$ and $C_{j}$. If the lower bound is positive, then $C_{i}⪯C_{j}$, otherwise, $C_{i}⪯̸C_{j}$.

### 6.2 Real-world data: Thailand's population household information

This dataset was obtained from Thailand household-population surveys from Thai government in 2018 [28]. The purpose of this survey was to analyze the Multidimensional Poverty Index (MPI) [29], [30], which is considered as a current main poverty index that the United Nations (UN) uses. We deployed the data of household incomes and careers information from 355,801 households of Khon Kaen province, Thailand to perform our analysis. We categorized careers of heads of households into 14 types: student (student), freelance (Freelance), plant farmer (AG-Farmer), peasant (AG-Peasant), orchardist (AG-Orchardist), fishery (AG-Fishery), animal farmer (AG-AnimalFarmer), unemployment (Unemployment), merchant (Merchant), company employee (EM-ComEmployee), business owner (Business-Owner), government's company employee (EM-ComOfficer), government officer (EM-Officer), and others (Others). The incomes in this dataset are annual incomes of households and the unit of incomes is in Thai Baht (THB).

Given a set of ordered pairs of career and household income, we analyzed the income gaps of different types of careers in order to study the inequality of population w.r.t. people careers.

### 6.3 Real-world data: NASDAQ Stock closing prices

The NASDAQ stock-market dataset has been obtained by the work in [4] from Yahoo! Finance.^1^ The dataset was collected from January 2000 to January 2016. It consists of a set of time series of stock closing prices of 1,060 companies. Each company time series has a total length as 4,169 time-steps. Due to the high variety of company sectors, in this study, we categorized these time series into five sectors: ‘Service & Life Style’, ‘Materials’, ‘Computer’, ‘Finance’, and ‘Industry & Technology’.

In order to observe dynamics of domination, we separated time series into two intervals: 2000-2014, and 2015-2016. For each interval, we aggregated the entire time series of a company using median.

Given a set of ordered pairs of closing-price median and sector, the purpose of this study is to find which sectors dominated others in each interval.

### 6.4 Parameter settings

We set a significant level α=0.05 and a number of times of sampling with replacement for a bootstrap approach is 1,000 for all experiments unless stated otherwise.

### 6.5 Running time and scalability analysis

**Figure 5:**
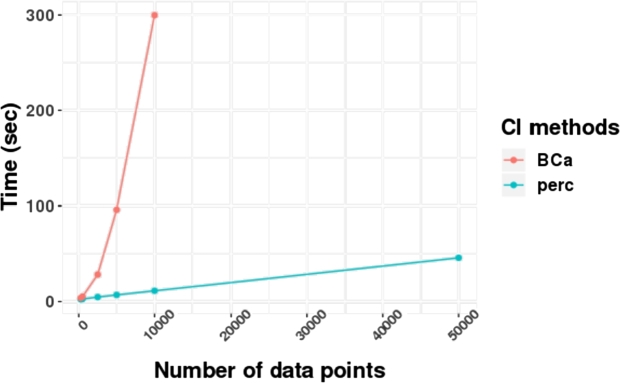
A comparison of running time between two methods of bootstrap confidence intervals with several numbers of data points.

In this experiment, we compared running times of two methods of bootstrapping to infer confidence intervals: BCa bootstrap (BCa) [16] and percentile (perc) approaches using simulation datasets from the previous section.^2^ We set a number of bootstrap replicates (numbers of times of sampling with replacement) at 4,000 rounds. In Fig. 5, the result implies that the BCa method was a lot slower than the percentile approach. In a dataset of 10,000 data points, the BCa bootstrap required the running time around 300 seconds while the percentile approach required only 11 seconds. Besides, for a dataset that has 500,000 data points, percentile approach was able to finish running around 11 minutes. This indicates that the percentile approach is scalable better than the BCa bootstrap.

**Figure 6:**
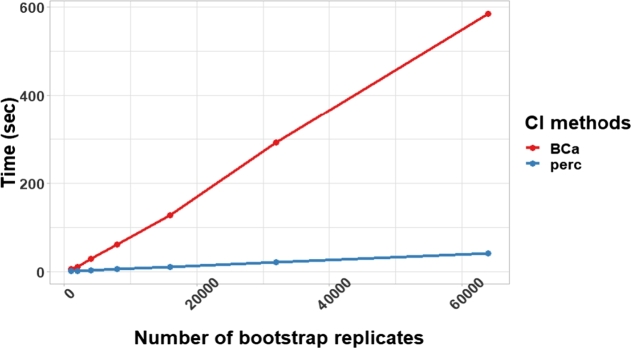
A comparison of running time between two methods of bootstrapping with different numbers of bootstrap replicates (numbers of times of sampling with replacement).

In an aspect of numbers of bootstrap replicates, Fig. 6 illustrates running times of two methods of bootstrapping with different numbers of bootstrap replicates.^3^ The BCa bootstrap required six times or more running time than percentile bootstrap (perc).

Lastly, when datasets are too large, one of common methods that can deal with a large dataset for inferring bootstrap confidence intervals is to sample some data points from a full dataset. Table 1 shows a result from both bootstrap methods using different numbers of data points sampling from a simulation dataset (40,000 data points with $p_{1}=0.1$) in Section 6.1.^4^ This result illustrates that a higher number of data points leads to a higher F1 score. In this dataset, we need only 20 percent of data points (8,000 data points) to accomplish a perfect F1 score at one for both bootstrap methods. However, the BCa method took longer running time than the perc method while both approaches provided almost similar F1 scores. Hence, for large datasets, we recommend users to use the percentile approach since it is fast and the performance is comparable or even better than the BCa method that we will show in the next section.

**Table 1:** A comparison of running time, numbers of data points, and F1 score between two methods of bootstrapping using a simulation dataset that has 40,000 data points. Each row represents a result from a specific number of data points sampling from the full dataset. F1 scores were computed w.r.t. a simulation ground truth in the task of categories ordering inference.

	Bootstrap: BCa		Bootstrap: perc	
#data points	F1 score	Time (sec)	F1 score	Time (sec)
400	0.67	9.16	0.40	6.40
4,000	0.67	27.50	0.89	10.01
8,000	1.00	64.61	1.00	13.15
20,000	1.00	242.22	1.00	22.60
40,000	1.00	838.30	1.00	37.61

## 7 Results

### 7.1 Simulation results

In this section, we report results of our analysis from simulation datasets (Section 6.1). The main task is an ordering inference; determining whether A⪯B for all pairs of categories.

Table 2 illustrates the categories ordering inference result. Each value in the table is the aggregate results of datasets from different values of $p_{1}$: $p_{1}=\{0.01,0.05,0.10,0.15,0.20,0.25,0.30,0.35,0.40\}$. The table shows that our approach (using Mann-Whitney) performance is better than all approaches. While ttest (pool.sd) performed the worst, the traditional t-test performed slightly better than both bootstrap approaches. Comparing between BCa and percentile bootstraps, the performance of percentile bootstrap is slightly better than the BCa bootstrap. Even though the BCa bootstrap covers the skew issue better than the percentile bootstrap [15], [16], our result indicates that percentile bootstrap is more accurate than the BCa bootstrap when the noise presents in the task of ordering inference.

**Table 2:** The categories ordering inference result; each approach is used to infer orders of any pair of two categories w.r.t. the real-values within each category.

	Precision	Recall	F1 scores
ttest (pool.sd)	0.61	0.52	0.55
ttest	0.72	0.72	0.72
Bootstrap: BCa	0.70	0.67	0.68
Bootstrap: Perc	0.73	0.68	0.70
EDOIF (Mann-Whitney)	0.77	0.85	0.81
Mean	0.60	1.00	0.75
Median	0.60	1.00	0.75

**Figure 7:**
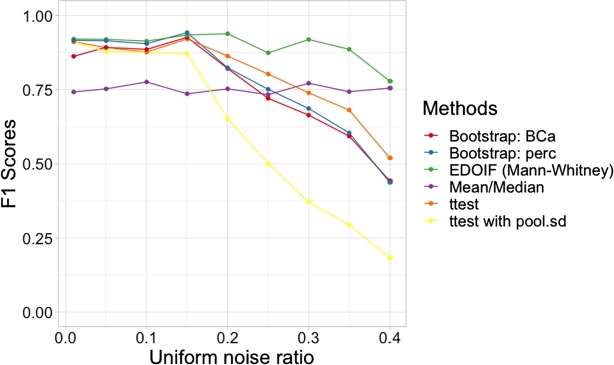
The sensitivity analysis of categories ordering inference. Simulation datasets containing different levels of noise were deployed for the experiment (best viewed in color codes).

Fig. 7 shows the result of sensitivity analysis of all approaches when the uniform noise presents in different degrees. The horizontal axis represents noise ratios and the vertical axis represents F1 scores in the task of ordering inference. According to Fig. 7, our approach (using Mann-Whitney) performed better than all methods in all levels of noise. t-test preformed slightly better than both bootstrap approaches. Results from Both bootstrap methods are quite similar. The t-test with (pool.sd) performed the worst. Both Table 2 and Fig. 7 illustrate the robustness of our approach.

We also compare our method with the summary statistics: mean and median to perform the categories ordering inference. Table 2 illustrates that mean and median had high recall but low precision values compared against other methods. This is due to the fact that when one distribution dominate other significantly, by using just simple summary statistics, we can detect the domination. However, when two distributions are not dominated each other, their means or medians might greater or lower than each other slightly due to the noise. This makes the false positive cases occur if we use these summary statistics to detect domination relations. Hence, the precision values of both mean and median are low. Fig. 7 also illustrates the sensitivity analysis results of summary statistics: mean and median. Even though the mean and median results were not affect by the degree of noise, they performed poorly compared to our approach (EDOIF). This makes the point that our method is more robust than summary statistics in this task.

### 7.2 Case study: ordering career categories based on Thailand's household incomes in Khon Kaen province

**Figure 8:**
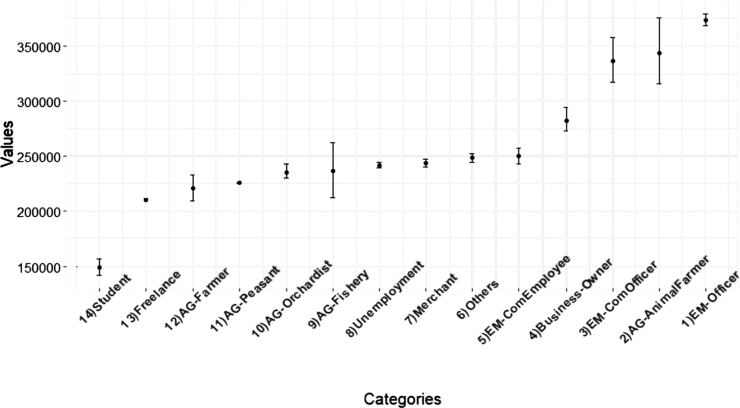
Confidence intervals of household incomes of the population from Khon Kaen province categorized by careers.

In this section, we report orders of careers based on incomes of a population in Khon Kaen province, Thailand. Due to the expensive cost of computation of the BCa bootstrap, in this dataset, since there are 353,910 data points, we used the percentile bootstrap as a main method. Fig. 8 illustrates the bootstrap-percentile confidence intervals of mean incomes of all careers with an order ascendingly sorted by income sample-means.

A government officer (EM-Officer) class is ranked as the 1st place of career that has the highest mean income, while a student class has the lowest mean income.

**Figure 9:**
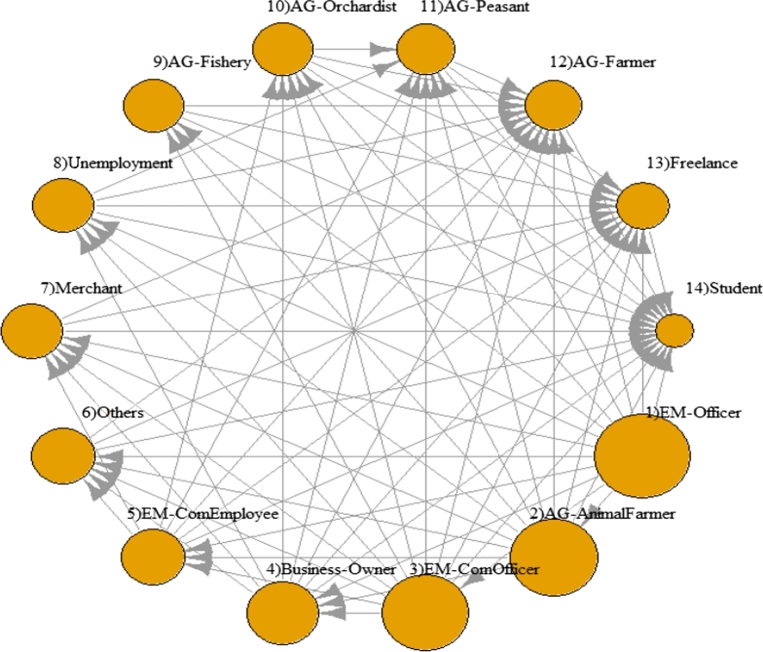
A dominant-distribution network of household incomes of the population from Khon Kaen province categorized by careers. A node size represents a magnitude of sample mean of incomes of a career.

**Figure 10:**
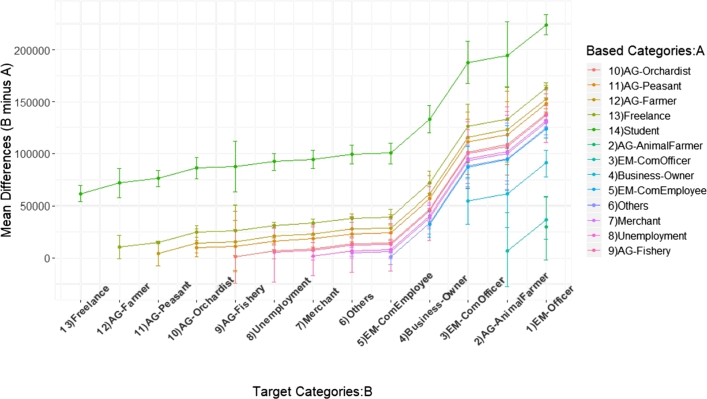
Mean-difference confidence intervals of career pairs based on household incomes of the population from Khon Kaen province.

Fig. 9 shows orders of dominant-distribution relations of career classes in a form of a dominant-distribution network. It shows that a government officer (EM-Officer) class dominates all career classes. In a dominant-distribution network, its network density represents a level of domination; higher network density implies there are many categories that are dominated by others. The network density of the network is 0.79. Since the network density is high, a higher-rank career class seems to dominate a lower-rank career class with high probability. This implies that different careers provide different incomes. In other words, gaps between careers are high. Fig. 10 provides the magnitudes of income-mean difference between pairs of careers in the form of confidence intervals. It shows us that the majority of pairs of different careers have gaps of annual incomes at least 25,000 THB (around $800 USD)!

Since one of definitions of economic inequality is income inequality [31], [32], [33], there is a high degree of career-income inequality in this area. In societies with a more equal distribution of incomes, people are healthier [32]. This inequality might lead to other issues such as health issue. Moreover, the income inequality is associate with happiness of people [33]. This case study shows that using our dominant-distribution network and mean-difference confidence intervals is a novel way of studying career-income inequality.

Table 3 shows the Khon Kaen empirical result of dominant-distribution network density inference varying numbers of data points sampling from 355,801 data points. Network densities of all methods increased when numbers of data points increased. This is due to a reason that when a number of samples is high, methods can distinguish whether one category dominates another better. Network densities of almost all methods are slightly different except ttest (pool.sd) that performed poorly in simulation datasets (Section 7.1).

**Table 3:** The Khon Kaen empirical result of network density inference varying numbers of data points sampling from 355,801 data points. Each data point represents an ordered pair of career and house-hold income of people in Khon Kaen province, Thailand. Each element in the table is a network density of a dominant-distribution network. Due to BCa's high cost of computation and limited resource, BCa was unable to perform on large datasets (N/A element).

#data points	ttest (pool.sd)	ttest	Boot: BCa	Boot: Perc	EDOIF (Mann-Whitney)
3539	0.09	0.36	0.43	0.40	0.47
7078	0.11	0.47	0.46	0.45	0.46
35391	0.22	0.69	N/A	0.66	0.70
176955	0.34	0.80	N/A	0.79	0.76
353910	0.36	0.87	N/A	0.82	0.79

In the aspect of using simple summary statistics, the network densities of domination networks in Table 3 cannot directly be derived from any simple summary statistics such as mean or median. This is because we have to infer whether one distribution is dominated by another efficiently before calculating the domination network and its related statistics. The simple mean or median performed poorly in this task (see the Section 7.1 for the performance of summary statistics). Additionally, the confidence intervals of mean difference in Fig. 10 also cannot derive by simply using mean or median since these summary statistics cannot be used to guarantee any lower or upper bound of the interval the same way as bootstrapping approaches do. In practice, knowing the confidence interval bounds make users know how much two systems are different from each other with high probability. By using mean or median, we know that whether two systems are different on average. However, we cannot claim anything that one system (distribution) dominates another with high probability. This makes the reliability of results difference when we use either simple summary statistics or bootstrapping approach like our method.

Specifically, Fig. 10 provides more reliable and informative results that whether two careers (e.g. students vs. freelance) are different and how much they are different with high probability. By using only difference of average income between two careers, we only know that whether they are different on average. However, we cannot claim whether the minimum income gaps of two careers are different with high probability. Only the 95%-mean-difference confidence intervals can tell us. For example, in case 1), AG-Farmer and Freelance have difference means, but the distributions of incomes of these two careers are not significantly different (w.r.t. our statistical testing and bootstrapping analysis). This implies that if we sampling two people from these two careers, we cannot conclude that a person from AG-Farmer has higher income than a person from freelance even though the income mean of AG-Farmer is higher than the freelance career. In contrast, in case 2), the students have significantly lower incomes than people from EM-Officers. Both careers have a large gap of mean and the high value of lower bound of the mean-difference-confidence-interval. The lower bound of mean-difference-confidence-interval tells us that if we sampling one student and one person from EM-Officers, then, with at least 95% of the times, a student has a lower income than an EM-officer at least 200k THB annually. Summary statistics like mean or median cannot distinguish between case 1 and case 2, but our approach can clearly distinguish them. The difference between case 1 and 2 is important for policies makers to provide support for any pairs of careers or studying income inequality. There is no income inequality in case 1, but the income inequality exists in case 2.

### 7.3 Case study: ordering aggregate-closing prices of NASDAQ stock market based on sectors

This case study reveals dynamics of sector domination in NASDAQ stock market. We report the patterns of dominate sectors that change over time in the market.

**Figure 11:**
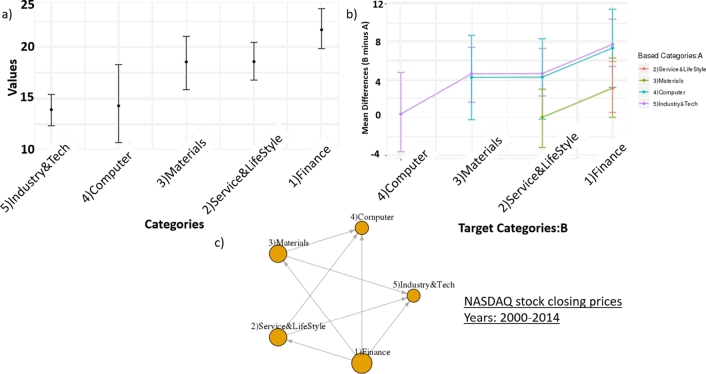
The sectors ordering result of NASDAQ stock closing prices from 1,060 companies between 2000 and 2014. a) Confidence intervals of closing prices of sectors. b) Confidence intervals of difference means of closing prices among sectors. c) A dominant-distribution network of sectors.

Fig. 11 shows the sectors ordering result of NASDAQ stock closing prices from 1,060 companies between 2000 and 2014. The dominated sector is ‘Finance’ sector that dominates all other sectors. Due to the high network density of the dominant-distribution network at 0.8, there are large gaps between sectors in this time interval.

**Figure 12:**
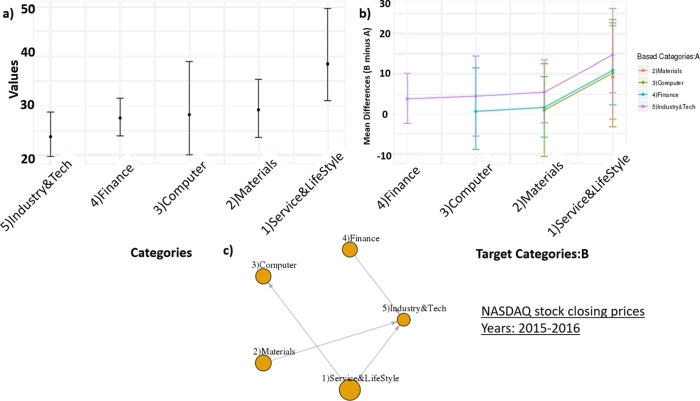
The sectors ordering result of NASDAQ stock closing prices from 1,060 companies between 2015 and 2016. We separated companies into five main sectors: ‘Service & Life Style’, ‘Materials’, ‘Computer’, ‘Finance’, and ‘Industry & Technology’. a) Confidence intervals of closing prices of sectors. b) Confidence intervals of difference means of closing prices among sectors. c) A dominant-distribution network of sectors.

On the other hand, in Fig. 12, the result of sectors ordering of NASDAQ stock closing prices between 2015 and 2016 demonstrates that there is no sector that dominates all other sectors. The network density is 0.4, which implies that the level of domination is less than the previous interval. The Finance sector is ranked as 4th position in the order. It is not because the Finance sector has a lower closing price in recent years, but all other sectors have higher closing prices lately. The computer sector has a higher closing price lately compared to the previous time interval, which is consistent with the current situation that the IT development (e.g. big data analytics, AI, blockchain) impacts many business scopes significantly [34].

**Figure 13:**
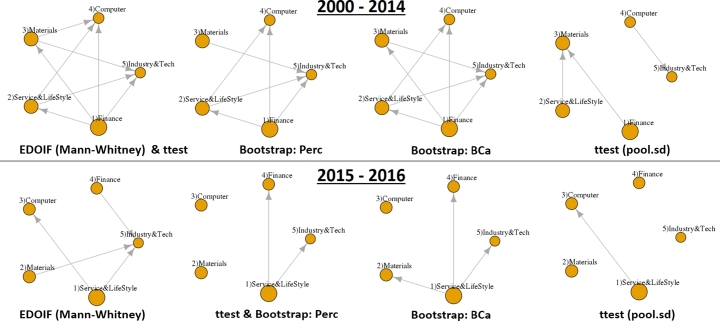
The empirical result of sectors ordering inference from NASDAQ stock closing prices. Dominant-distribution networks were inferred from 1,060 companies using two intervals: (top) from 2000 to 2014 and (bottom) from 2015 to 2016.

Fig. 13 shows the empirical result of sectors ordering inference from NASDAQ stock closing prices. In an interval from 2000 to 2014, all methods have a high numbers of domination edges (except ttest (pool.sd) that performed poorly in simulation datasets (Section 7.1).) In contrast, from 2015 to 2016, there are few edges in dominant-distribution networks from all methods.

This result indicates that almost all methods reported the same dynamics of NASDAQ stock closing prices from the interval that has a high degree of domination (2000-2014) to the interval that has a lower degree of domination (2015-2016).

## 8 Conclusion

In this paper, we proposed a framework that is able to infer orders of categories based on their expectation of real-number values using the estimation statistics. Not only reporting whether an order of categories exists, but our framework also reports a magnitude of difference of each consecutive pairs of categories in the order using confidence intervals and a dominant-distribution network.

In large datasets, our framework is scalable well using the percentile bootstrap approach compared against the existing framework, DABESTR, that uses the BCa bootstrap. The proposed framework was applied to two real-world case studies: 1) ordering careers based on 350,000 household incomes from the population of Khon Kaen province in Thailand, and 2) ordering sectors based on 1,060 companies' closing prices of NASDAQ stock market between years 2000 and 2016.

The results of careers ordering showed income-inequality among different careers in a dominant-distribution network. The stock market results illustrated dynamics of sectors that dominate the market can be changed over time.

The encouraging results show that our approach is able to be applied to any other research area that has category-real ordered pairs. Our proposed *Dominant-Distribution Network* provides a novel approach to gain new insight of analyzing category orders. The software of this framework is available for researchers or practitioners with a user-friendly R package on R CRAN at [7].

## Declarations

### Author contribution statement

C. Amornbunchornvej: Conceived and designed the experiments; Performed the experiments; Analyzed and interpreted the data; Contributed reagents, materials, analysis tools or data; Wrote the paper.

N. Surasvadi: Analyzed and interpreted the data; Contributed reagents, materials, analysis tools or data; Wrote the paper.

A. Plangprasopchok: Contributed reagents, materials, analysis tools or data; Wrote the paper.

S. Thajchayapong: Analyzed and interpreted the data; Wrote the paper.

### Funding statement

This paper was supported in part by the Thai People Map and Analytics Platform (TPMAP), a joint project between the office of National Economic and Social Development Council (NESDC) and the 10.13039/501100011058National Electronics and Computer Technology Center (NECTEC), which is an organization under the National Science and Technology Development Agency (NSTDA), Thailand. The grant number is P1852296.

### Declaration of interests statement

The authors declare no conflict of interest.

### Additional information

No additional information is available for this paper.

## Footnotes

1 http://finance.yahoo.com/.

2 The computer specification that we used in this experiment is Dell 730, with CPU Intel Xeon E5-2630 2.4 GHz, and Ram 128 GB.

3 The dataset has 2,500 data points.

4 We set a number of bootstrap replicates at 40,000 for all cases in the table.
